# Patient‐reported reasons for declining same‐day antiretroviral therapy initiation in routine HIV care settings in Lusaka, Zambia: results from a mixed‐effects regression analysis

**DOI:** 10.1002/jia2.25560

**Published:** 2020-07-03

**Authors:** Jake Pry, Jenala Chipungu, Helene J Smith, Carolyn Bolton Moore, Jacob Mutale, Miquel Duran‐Frigola, Theodora Savory, Michael E Herce

**Affiliations:** ^1^ Implementation Science Unit Centre for Infectious Disease Research in Zambia (CIDRZ) Lusaka Zambia; ^2^ Division of Infectious Diseases School of Medicine Washington University St. Louis MO USA; ^3^ School of Medicine University of Alabama Birmingham AL USA; ^4^ Joint IRB‐BSC‐CRG Program in Computational Biology Institute for Research in Biomedicine (IRB Barcelona) The Barcelona Institute of Science and Technology Barcelona Catalonia Spain; ^5^ Institute for Global Health & Infectious Diseases School of Medicine University of North Carolina Chapel Hill NC USA

**Keywords:** Same‐day ART, Zambia, HIV care continuum, Africa < Region, testing Stigma, ARV, linkage to care

## Abstract

**Introduction:**

In the current “test and treat” era, HIV programmes are increasingly focusing resources on linkage to care and same‐day antiretroviral therapy (ART) initiation to meet UNAIDS 95‐95‐95 targets. After observing sub‐optimal treatment indicators in health facilities supported by the Centre for Infectious Disease Research in Zambia (CIDRZ), we piloted a “linkage assessment” tool in facility‐based HIV testing settings to uncover barriers to same‐day linkage to care and ART initiation among newly identified people living with HIV (PLHIV) and to guide HIV programme quality improvement efforts.

**Methods:**

The one‐page, structured linkage assessment tool was developed to capture patient‐reported barriers to same‐day linkage and ART initiation using three empirically supported categories of barriers: social, personal and structural. The tool was implemented in three health facilities, two urban and one rural, in Lusaka, Zambia from 1 November 2017 to 31 January 2018, and administered to all newly identified PLHIV declining same‐day linkage and ART. Individuals selected as many reasons as relevant. We used mixed**‐**effects logistic regression modelling to evaluate predictors of citing specific barriers to same‐day linkage and ART, and Fisher’s Exact tests to assess differences in barrier citation by socio‐demographics and HIV testing entry point.

**Results:**

A total of 1278 people tested HIV positive, of whom 126 (9.9%) declined same‐day linkage and ART, reporting a median of three barriers per respondent. Of these 126, 71.4% were female. Females declining same‐day ART were younger, on average, (median 28.5 years, interquartile range (IQR): 21 to 37 years) than males (median 34.5 years, IQR: 26 to 44 years). The most commonly reported barrier category was structural, “clinics were too crowded” (n = 33), followed by a social reason, “friends and family will condemn me” (n = 30). The frequency of citing personal barriers differed significantly across HIV testing point (χ^2^ *p* = 0.03). Significant predictors for citing ≥1 barrier to same‐day ART were >50 years of age (OR: 12.59, 95% CI: 6.00 to 26.41) and testing at a rural facility (OR: 9.92, 95% CI: 4.98 to 19.79).

**Conclusions:**

Given differences observed in barriers to same‐day ART initiation reported across sex, age, testing point, and facility type, new, tailored counselling and linkage to care approaches are needed, which should be rigorously evaluated in routine programme settings.

## 1 INTRODUCTION

Treatment programmes are increasingly focusing resources to meet the UNAIDS “second 95” target such that 95% of all people diagnosed with HIV receive antiretroviral therapy (ART) [1]. In Zambia, opt‐out HIV testing services (HTS) and same‐day ART have been widely implemented in Ministry of Health (MOH) facilities as part of the national “test and treat” strategy to increase HIV status awareness and ART coverage [2]. Despite these efforts, suboptimal linkage to care and delays with ART initiation threaten progress towards the second 95 as demonstrated by recent test and treat trials from Zambia and other sub‐Saharan African countries [3, 4, 5].

Although a body of research since the advent of ART scale up has documented barriers and facilitators to linkage to care [6, 7, 8, 9], less is known about these factors in the current treat all era [10]. Recent qualitative data from the HPTN 071 (PopART) study suggest that a confluence of diverse patient (e.g. lack of trust in HIV test results), community (e.g. promotion of alternative treatments) and health system factors (e.g. protracted ART initiation procedures resulting from consolidating many pre‐ART visits into one) affect linkage to care and rapid ART initiation in new ways since the dawn of test and treat [5]. While these and other trial findings provide insights for improving linkage to care, scarce data describe reasons for untimely linkage to care and poor same‐day ART uptake within routine HIV treatment programmes in high‐burden settings like Zambia. Moreover, few studies have examined patient‐reported barriers for declining same‐day linkage and ART initiation at the time of HIV diagnosis [11, 12, 13, 14, 15, 16].

Following Zambia’s introduction of test and treat in December 2016, we observed sub‐optimal rates of linkage to care and ART initiation (as low as 72%) in MOH facilities supported by the Centre for Infectious Disease Research in Zambia (CIDRZ) in the capital of Lusaka [17]. In response, we piloted a brief “linkage assessment” tool to: (1) specify and quantify barriers to ART initiation among newly identified PLHIV declining treatment on the day of HIV diagnosis; and (2) inform programme quality improvement efforts at CIDRZ‐supported health facilities. In this paper, we describe the most frequently cited patient‐reported barriers to same‐day ART initiation in a “real world,” programmatic setting and examine associations with patient demographic characteristics, HIV testing point and facility type with the goal of tailoring linkage interventions to address the identified barriers.

## 2 METHODS

### 2.1 Population

The study population included all adult patients (≥18 years) who presented for routine HTS in one of three health facilities in Lusaka Province, Zambia between 1 November 2017 and 31 January 2018; had a documented positive screening and confirmatory HIV test result; and indicated to clinic staff their intention to decline linkage to care and same‐day ART initiation.

### 2.2 Setting

We designed, piloted and integrated a one‐page, paper‐based linkage assessment tool within routine HTS settings to identify reasons for declined or delayed linkage to care and ART initiation. We purposively selected three health facilities from among 56 CIDRZ‐supported facilities in Lusaka Province to: reflect each of the three main types of public‐sector health facilities (i.e. rural primary health centre, urban primary health centre and urban first‐level hospital) receiving support from CIDRZ and other PEPFAR implementing partners following introduction of test and treat nationally; and capture reasons at high‐volume sites (i.e. facilities with an average of >250 individuals receiving HTS per quarter in 2017) with low linkage and same‐day ART initiation in the routine HIV treatment programme. Within each facility, the linkage assessment tool was administered by trained HTS counsellors during routine post‐test counselling to facilitate individualized counselling tailored to patient‐reported barriers. Estimated HIV prevalence in Lusaka province was approximately 16% at the time of the pilot [18].

### 2.3 Measurements

The linkage assessment tool contained items informed by an empirically supported questionnaire previously implemented during a CIDRZ loss to follow‐up study described elsewhere [19]. Reasons and thematic categories for refusal were selected based on published literature from Zambia and the region [12, 19, 20]. Aligned with this evidence and the social ecological framework, 22 pre‐set reasons for refusal were grouped under three main categories of barriers – personal (10 reasons), social (5 reasons) and structural (7 reasons) [21]. During post‐test counselling, patients were asked to report as many barriers (across all barrier categories) as they felt applied to their own decision to decline same‐day linkage to care and ART initiation. We then abstracted routinely reported data on age, sex, health facility level and HIV testing entry point for all patients testing HIV positive during the review period.

### 2.4 Analysis

We used descriptive statistics to summarize the frequency of barriers cited and chi‐squared tests to evaluate differences in barrier category by testing entry point, age and sex among those who declined same‐day linkage and ART. Proportional Venn diagrams illustrate the relative distribution of reasons for declining same‐day ART initiation. Odds ratios for declining same‐day ART initiation were modelled using mixed‐effects logistic regression allowing random effects for health facility and fixed effects for all available predictors including age, sex, health facility level and HIV testing point. Predictive probabilities were estimated using our mixed‐effects regression model including interaction between sex and age by specific barrier. All analyses were developed using Stata 15.1 SE (StataCorp, College Station, TX, USA) and figures were refined using R software v3.6.1 (R Foundation for Statistical Computing, Vienna, Austria) [22].

### 2.5 Ethics statement

The study protocol was approved by the U.S. Centers for Disease Control & Prevention (2018‐381), University of Zambia Biomedical Research Ethics Committee (011‐12‐17), University of North Carolina at Chapel Hill, USA (18‐0854) and the Institutional Review Board at Washington University, St. Louis, USA (2019‐11143) without requiring patient consent for review of de‐identified, routinely collected data.

## 3 RESULTS AND DISCUSSION

Among 16,323 people who accessed HTS across three facilities during the evaluation period, 1,278 (7.8%) individuals had a positive HIV test result, of whom 62.1% were female and 126 (9.9%) declined same‐day linkage and ART (Table 1). Only 3.5% of those testing positive in the youngest age‐band (<25 years) declined same‐day ART initiation, whereas 31.6% of those in the eldest age‐band (>50 years) declined. A higher proportion of patients received HTS in a rural clinic setting among those who declined same‐day ART initiation (14.3%) than among those who did not decline same‐day linkage and ART (1.8%).

**Table 1:** Characteristics of population testing HIV positive in routine, facility‐based HIV testing services (N = 1278)

Factor	Level	Did not decline n (%) N = 1152	Declined n (%) N = 126	*p*‐value
Sex	Female	704 (61.1%)	90 (71.4%)	0.02
	Male	448 (38.9%)	36 (28.6%)	
Age category	<25	300 (26.0%)	11 (8.7%)	<0.01
	25 to 34	404 (35.1%)	45 (35.7%)	
	35 to 50	383 (33.2%)	40 (31.7%)	
	>50	65 (5.6%)	30 (23.8%)	
Facility	First level hospital	602 (52.3%)	52 (41.3%)	<0.01
	Urban clinic	529 (45.9%)	56 (44.4%)	
	Rural clinic	21 (1.8%)	18 (14.3%)	
Entry point	PITC	680 (59.0%)	83 (68.6%)	<0.01
	VCT	263 (22.8%)	38 (31.4%)	
	Index testing	14 (1.2%)	0 (0.0%)	
	TB	40 (3.5%)	0 (0.0%)	
	PMTCT	155 (13.5%)	0 (0.0%)	

Categories not summing to the specified N are an artefact of missing/unavailable data.

PITC, provider initiated testing and counselling; PMTCT, prevention of mother‐to‐child transmission of HIV clinic; TB, tuberculosis clinic; VCT, voluntary counselling and testing.

**Figure 1:**
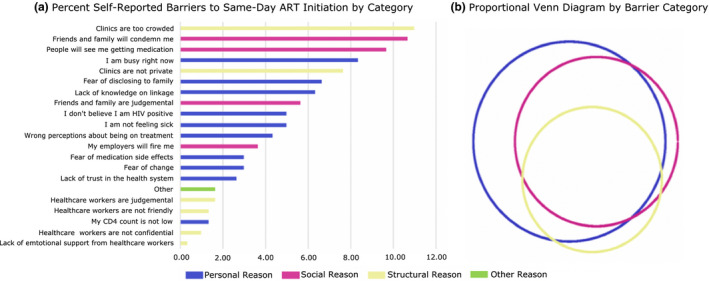
**(a)** Barrier cited by category **(b)** proportional Venn diagram by barrier category (N = 126).

There were 300 specific barriers captured by 126 individuals declining same‐day ART initiation. Personal barriers were most commonly cited (87.3%) and many (34.9%) reported at least one barrier in all three barrier categories, supporting previous findings on the multi‐level nature of the process of linkage to care (Figure 1b) [5, 10]. The single most commonly cited barrier was in the structural category, namely “Clinics are too crowded” (11.0%), followed by social barriers, “Friends and family will condemn me” (10.7%) and “People will see me getting medication” (9.7%) (Figure 1a). The most common combination of barriers among those that reported >1 barrier (60.3%) was: “Clinics are too crowded” (structural), “People will see me picking up my medication” (social) and “Lack of knowledge about linkage” (personal). Another common combination of responses, among those who reported >1 barrier was as follows: “Fear of disclosing [HIV status] to family” (personal), “Friend and family will condemn me” (social) and “Clinics are not private” (structural).

Reports of worry about being seen in over‐crowded clinics align with literature on the substantial barriers to care posed by stigma and health service factors like waiting times, and highlight the need for clinics to provide patient‐centred care and keep visits private [5, 10, 23]. While it can be difficult to pragmatically address such structural barriers, particularly in resource‐limited settings, several emerging approaches may help, including clinic migration and decentralization [24]. Another promising method of addressing structural barriers may be the continued scale up of differentiated service delivery (DSD) models, including community adherence groups, urban adherence groups and fast‐track models, all of which may expedite HIV services, decongest crowded health facilities and improve clinic flow for patients [25, 26, 27, 28, 29, 30, 31].

**Figure 2:**
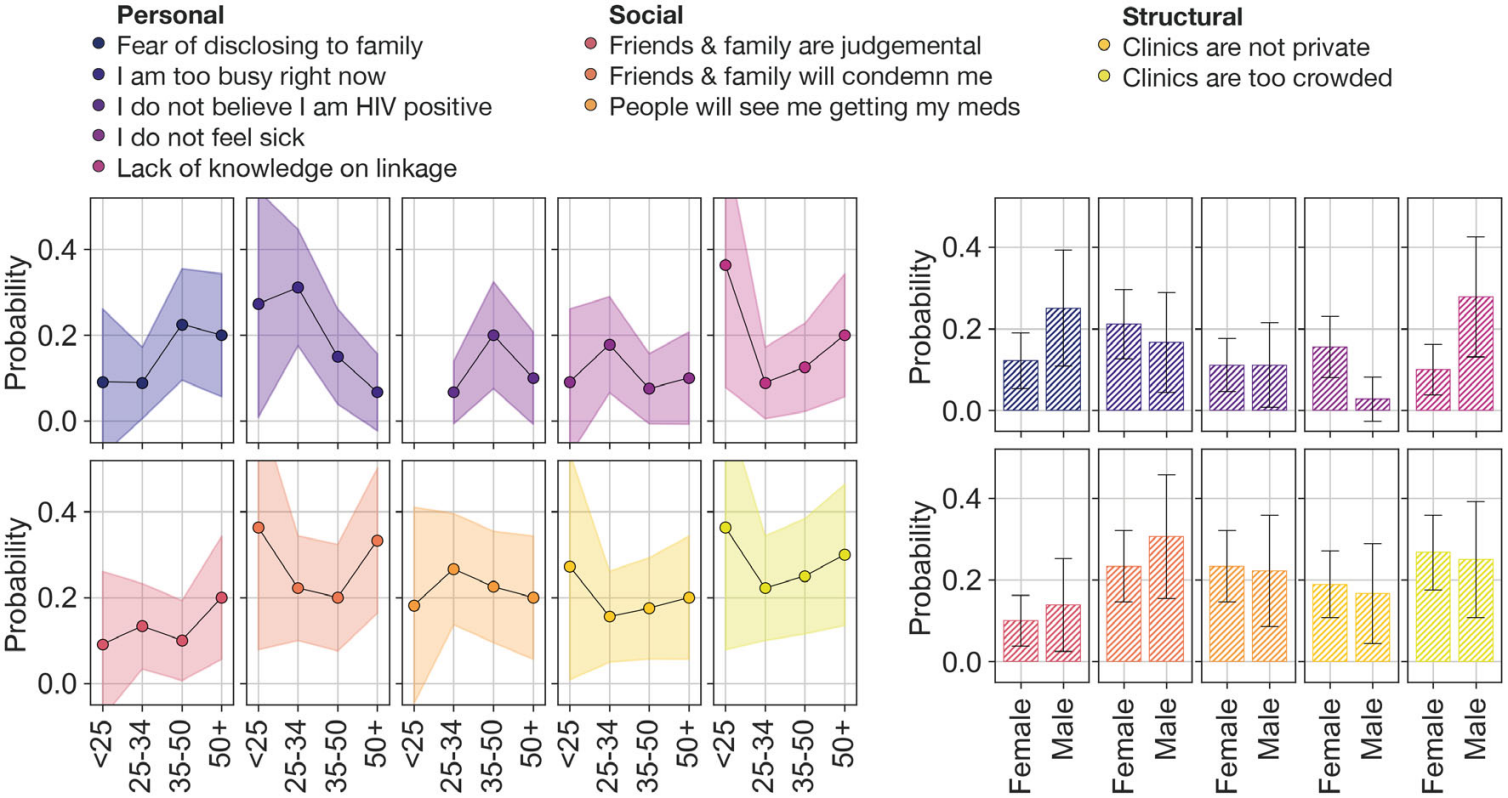
Probability of citing barrier by age category and sex for the ten most frequently reported barriers (N = 126). Adjusted by age, sex and clinic; predictive probabilities were derived from a mixed‐effects model including a random effect for facility and fixed effects for both age and sex.

We observed differences in cited reasons by both sex and age (Figure 2). For example PLHIV under age 35 were more likely to report being too busy to complete same‐day ART initiation, whereas those under age 25 noted a lack of knowledge about the benefits of same‐day linkage to care and ART initiation. The predictive probability for disclosing to family members was higher for men than women, and women had a higher predictive probability to cite not feeling sick. Taken together, these findings suggest a need for interventions that are tailored to individual demographic groups, including the needs and preferences of young men and women.

Somewhat surprisingly, we found that those testing through VCT were significantly more likely to report a personal barrier to initiation (92.1%) than those accessing testing through PITC (75.0%) (Mann–Whitney *p* = 0.03). While VCT clients presumably have more awareness of their HIV risk when seeking out HTS, that may not necessarily translate into motivation to initiate ART if found HIV positive. As such, there may be opportunities to tailor existing post‐test counselling and individualized support approaches according to HIV testing entry point [32]. For some entry points, like PMTCT, substantial evidence already exists for tailored approaches to promote timely linkage and ART uptake due to more programmatic experience with test and treat (inaugurated by Option B+) [33]. However, newer testing entry points, like index testing, require further study to identify differentiated approaches to optimize timely ART initiation.

Important behaviour change communication around “undetectable equals untransmittable” (U = U) has yet to be fully incorporated into the counselling curriculum in Zambia [34]. Personal barriers such as “Wrong perceptions about being on treatment” could potentially be ameliorated by incorporating vital health messaging such as “undetectable equals untransmittable” (U = U) into post‐test counselling [34]. Adequate time for reflection and consideration of such messaging may be needed by some patients to spark behaviour change, and, ultimately, timely uptake of ART [5, 33].

Receptiveness to same‐day linkage and ART may be improved through more patient‐centred approach(s) to HIV care delivery in ART clinics [10]. This is evidenced by many of the structural barriers identified, including: “Healthcare workers are judgmental,” “Healthcare workers are not friendly,” “Healthcare workers are not confidential” and “Lack of emotional support from healthcare workers.” Interestingly, these barriers were cited by patients prior to ART initiation procedures, and often before interacting with healthcare workers from clinical departments, suggesting that previous negative experiences with the healthcare system or prevailing negative sentiments about ART care in the community adversely affect patient perspectives and expectations. Relatedly, improved communication between patients and healthcare workers could positively impact personal barriers such as “Lack of knowledge on linkage,” and could help mitigate social barriers like “My employer will fire me” through provision of extended, multi‐month prescriptions of ART. Generally, patient‐centred care models may have powerful effects on same‐day ART initiation through mitigation of commonly cited barriers [35, 36].

Our adjusted mixed‐effects regression model identified sub‐groups who may be more likely to decline same‐day linkage and ART. PLHIV > 50 years of age had over 12 times the odds of refusing same‐day linkage and ART (OR: 12.59; 95% CI: 6.00 to 26.41) as those less than 25 years of age (Table 2). Men were significantly less likely to decline same‐day ART initiation compared to women (OR: 0.63; 95% CI: 0.42 to 0.94). Interestingly, those testing at a rural clinic had significantly higher odds of declining same‐day linkage and ART compared to those in an urban first‐level hospital (OR: 9.92; 95% CI: 4.98 to 19.79). These findings reinforce the concept that willingness to immediately initiate ART may differ by gender, age and local context and suggest a need for granular age, sex and site disaggregation of linkage to care and ART uptake data to fully evaluate and optimize routine programme performance [5].

**Table 2:** Factors associated with refusing same‐day ART initiation (N = 1278)

Variable	Level	Unadjusted			Adjusted		
		OR	*p*‐value	95% CI	OR	*p*‐value	95% CI
Age	<25	Ref.	Ref.	Ref.	Ref.	Ref.	Ref.
	25 to 34	3.04	0.01	(1.55 to 5.97)	2.93	<0.01	(2.03 to 4.22)
	35 to 50	2.85	<0.01	(1.44 to 5.65)	3.04	0.02	(1.15 to 7.99)
	>50	12.59	<0.01	(6.00 to 26.41)	14.08	<0.01	(4.33 to 45.75)
Sex	Female	Ref.	Ref.	Ref.	Ref.	Ref.	Ref.
	Male	0.63	0.024	(0.42 to 0.94)	0.40	<0.01	(0.34 to 0.63)
Clinic	Urban first Level Hospital	Ref.	Ref.	Ref.	Ref.	Ref.	Ref.
	Urban clinic	1.23	0.31	(0.83 to 1.82)	1.12	<0.01	(1.04 to 1.21)
	Rural clinic	9.92	<0.01	(4.98 to 19.79)	14.97	<0.01	(7.51 to 29.85)
Entry Point	PITC	Ref.	Ref.	Ref.	Ref.	Ref.	Ref.
	VCT	1.18	0.42	(0.79 to 1.78)	1.51	0.80	(0.06 to 35.30)

Adjusted estimates model includes all covariates presented in table. CI, confidence interval; OR, odds ratio; PITC, Provider Initiated Testing and Counselling; VCT, Voluntary Counselling and Testing.

### 3.1 Limitations

Our linkage assessment tool was designed to be used in routine HTS settings, and, as such, we encountered several limitations in its implementation. First, we were unable to identify the primary barrier that patients reported for declining same‐day linkage and ART initiation. Use of a tablet‐based tool may have enabled ranking of patient‐reported barriers. Second, we were unable to verify whether and when PLHIV started ART subsequent to declining immediate linkage to care and ART. Ongoing programmatic efforts to electronically link HTS and ART records may better facilitate this process. Third, barrier categories may not have been fully disjoint, and reasons attributed to different categories may reflect alternative ways of describing the same underlying phenomenon; results of an ongoing qualitative analysis are intended to explore such phenomena. Finally, we were only able to pilot the tool in three health facilities and to evaluate reason in a small proportion of newly diagnosed PLHIV in Lusaka, Zambia, limiting the generalizability of our findings. Thus, while our findings may not be fully generalizable to all HIV programmatic settings in Zambia, we were careful to select facilities generally representative of those in the public sector in Lusaka, and note that many of the trends and observations we observed were consistent with those seen in multi‐site pragmatic trials [5].

## 4 CONCLUSIONS

Given the differences observed in patient‐reported reasons for declining same‐day ART initiation across sex, age, facility level and testing entry point, new, tailored post‐test counselling and linkage to care support approaches are needed, which should be rigorously evaluated in routine programmatic settings. Such approaches can include longitudinal strength‐based counselling and DSD models, which could be adapted and scaled to help meet the unique needs of newly diagnosed PLHIV. These approaches should provide services starting at the time of HIV diagnosis and should function to decompress busy clinics to better support patients with rapid ART initiation, and address personal and social barriers relating to stigma, discrimination and HIV status disclosure.

## COMPETING INTEREST

All authors: No conflicts.

## AUTHORS’ CONTRIBUTIONS

JP lead in writing and all analysis. JC guided development of the linkage assessment tool. HS directed implementation of the linkage assessment tool. CB directs underlying programme and assisted the development of implementation plan for linkage assessment tool. JM provided assistance in the procurement and curation of analysis datasets. MF provided assistance in data analysis and illustration. TS director of programme and assisted in identification of linkage failure and subsequent development of linkage assessment tool. MH substantially contributed to writing and assisted with development of the linkage assessment tool and pilot implementation. All authors have read and approved the final manuscript.
